# Microbicidal effects of weakly acidified chlorous acid water against feline calicivirus and *Clostridium difficile* spores under protein-rich conditions

**DOI:** 10.1371/journal.pone.0176718

**Published:** 2017-05-04

**Authors:** Hisataka Goda, Hitoshi Yamaoka, Haruyuki Nakayama-Imaohji, Hiroyuki Kawata, Isanori Horiuchi, Yatsuka Fujita, Tamiko Nagao, Ayano Tada, Atsushi Terada, Tomomi Kuwahara

**Affiliations:** 1 Honbu Sankei Co. Ltd., 2-2-53 Shiromi, Chuou-ku, Osaka, Japan; 2 Department of Microbiology, Faculty of Medicine, Kagawa University, 1750–1 Miki, Kagawa, Japan; 3 Faculty of Nursing, Shikoku University, Furukawa, Ojin-cho, Tokushima, Japan; 4 Department of Veterinary Medicine, Nihon University, Kameino, Fujisawa, Japan; Tallinn University of Technology, ESTONIA

## Abstract

Sanitation of environmental surfaces with chlorine based-disinfectants is a principal measure to control outbreaks of norovirus or *Clostridium difficile*. The microbicidal activity of chlorine-based disinfectants depends on the free available chlorine (FAC), but their oxidative potential is rapidly eliminated by organic matter. In this study, the microbicidal activities of weakly acidified chlorous acid water (WACAW) and sodium hypochlorite solution (NaClO) against feline calcivirus (FCV) and *C*. *difficile* spores were compared in protein-rich conditions. WACAW inactivated FCV and *C*. *difficile* spores better than NaClO under all experimental conditions used in this study. WACAW above 100 ppm FAC decreased FCV >4 log_10_ within 30 sec in the presence of 0.5% each of bovine serum albumin (BSA), polypeptone or meat extract. Even in the presence of 5% BSA, WACAW at 600 ppm FAC reduced FCV >4 log_10_ within 30 sec. Polypeptone inhibited the virucidal activity of WACAW against FCV more so than BSA or meat extract. WACAW at 200 ppm FAC decreased *C*. *difficile* spores >3 log_10_ within 1 min in the presence of 0.5% polypeptone. The microbicidal activity of NaClO was extensively diminished in the presence of organic matter. WACAW recovered its FAC to the initial level after partial neutralization by sodium thiosulfate, while no restoration of the FAC was observed in NaClO. These results indicate that WACAW is relatively stable under organic matter-rich conditions and therefore may be useful for treating environmental surfaces contaminated by human excretions.

## Introduction

Excretions from symptomatic or asymptomatic carriers of pathogens are the major sources of infectious diseases [1, 2]. Environmental surfaces contaminated by these excretions are often involved in outbreaks of norovirus and multi-drug resistant microorganisms [3–5]. Therefore, routine cleaning and disinfection of environmental surfaces as well as hand washing of workers in healthcare facilities or food plants is an important measure to block pathogen transmission [6–12]. In particular, facilities and plants, including seats and toilets, shared with a number of people should be adequately sanitized to prevent transmission of pathogens [13, 14].

Additionally, healthcare workers are often required to rapidly and accurately disinfect environmental surfaces contaminated by human excretions such as sudden vomit in norovirus gastroenteritis or recurring diarrhea in *Clostridium difficile* infection. Because these microorganisms are resistant to commonly and routinely used disinfectants such as quaternary ammonium and alcohol-based antiseptics [3], healthcare workers face difficulties in choosing the appropriate sanitizer. Restaurants and food factories, where norovirus food poisoning occurs frequently, face a similar situation.

Chlorine is a key agent in inactivating non-enveloped viruses and spore-forming bacteria [15, 16]. Authorized guidelines recommend the disinfection of environmental surfaces contaminated by norovirus and *C*. *difficile* with high concentrations of sodium hypochlorite (NaClO) [15, 16]. The microbicidal mechanism of chlorine is oxidative damage to the functional molecules in microorganisms such as DNA or proteins [17–19]. Therefore, the microbicidal effects of chlorine-based sanitizers are dependent on their oxidative potential. The oxidative potential of chlorine is determined by free available chlorine (FAC), which indicates the level of reactive chlorine contributing to oxidation [20, 21], while the total chlorine (TC) level measured by iodometric titration methods [22] indicates all of the chlorine, including the less reactive combined chlorine. In the case of NaClO, total chlorine is equivalent to FAC because all of the chlorine (HClO, ClO^-^) is involved in nucleophilic reactions.

Chlorous acid (HClO_2_)-based sanitizers such as acidified sodium chlorite solution (ASC) and weakly acidified chlorous acid water (WACAW) have been applied to food and environmental sanitation [23–26]. HClO_2_-based sanitizers have been reported to be more stable than NaClO under organic-matter-rich conditions [24, 26, 27]. They contain various forms of chlorinated oxides such as HClO_2_, dissolved ClO_2_ and ClO_2_^-^, and the former two are responsible for most of the microbicidal activity of WACAW. The predominant species in chlorous acid solutions above pH 4.0 (as in WACAW, pH 5.0–6.0) are ClO_2_^-^ and HClO_2_ [28]. Of these, HClO_2_ plays a major role in microbial killing of WACAW since oxidizing potential of ClO_2_^-^ is much weaker than HClO_2_ or ClO_2_ [29]. Total chlorine (TC) level of WACAW is much higher than FAC level, indicating that ClO_2_^-^ is a major chlorinated oxide species in this sanitizer before use.

In general, the microbicidal effect of chlorine-based sanitizer is assessed by FAC, not TC, level. Therefore, the FAC levels of the test chlorine solutions should be adjusted in comparative studies on microbicidal effects or stability. *N*,*N*-diethyl-*p*-phenylenediamine sulfate (DPD) is usually utilized to monitor the FAC level of chlorine solution because this indicator is less oxidized by combined chlorine [30].

In this study, the microbicidal activities of WACAW and NaClO at various FAC levels were compared in protein-rich solutions containing human norovirus surrogate, feline calicivirus (FCV) or *C*. *difficile* spores in order to simulate the disinfection of human excretions containing these important nosocomial pathogens.

## Materials and methods

### Chemicals

The sanitizers used in this study were chlorous acid water (Honbusankei Co., Ltd.) and NaClO (Nankai Chemical Co., Ltd.). Sodium thiosulfate, sodium chlorite, KH_2_PO_4_, K_2_HPO_4_, NaOH, *N*,*N*-diethyl-*p*-phenylenediamine (DPD) and sodium sulfate were purchased from Wako Pure Chemical Industries, Ltd. 3,3',5,5'-tetramethylbenzidine (TMB) was purchased from Tokyo Chemical Industry Co. Ltd. BSA (35% in PBS), polypeptone and meat extract were purchased from Sigma, Sumitomo Dainippon Pharma Co., Ltd. and Nacalai Tesque Inc., respectively. BSA, polypeptone and meat extract were added to microbicidal assays as an organic matter load.

### Measurement of TC

The TC levels of NaClO and WACAW were measured by an iodometric titration method [22]. The test solutions were diluted 10-fold with ion-exchanged water and mixed with 10% (w/w) potassium iodine and 10% (w/w) sulfate. After 15 min at room temperature in the dark, 0.1 M sodium thiosulfate solution was added until the color of the mixture turned light yellow. After starch solution (1%, w/w) was added, the mixtures were titrated with 0.1 M sodium thiosulfate solution until they became clear. The total chlorine (R) was calculated using the following equation with the weight of added sodium thiosulfate (A), factor of sodium thiosulfate (F), weight of sample used (W) and dilution rate of sample (K):

R=(A×F/W)x0.0035x100xKx10,000

### Measurement of FAC levels

The FAC levels (1–5,000 ppm (w/v)) of WACAW or NaClO were adjusted using the DPD method. One gram of ground DPD was mixed with 24 g sodium sulfate anhydrate, which was used as an indicator. After the test sample (9.5 ml) was buffered with 0.5 ml of 0.2 M KH_2_PO_4_ (pH 6.5), 0.1 g of DPD including sodium sulfate anhydrate was added, and the absorbance at 510 nm was measured. FAC levels of WACAW were calculated from standard curves generated with NaClO. The FAC levels of the test sanitizers after contact with organic substances were monitored by TMB method as previously described [27]. Combined chlorine level was calculated by subtracting FAC from TC.

### Preparation of sanitizers

ASC was prepared by acidifying the sodium chlorite solution of 2,400 ppm TC to pH 2.3 with 0.1 M HCl. WACAW was prepared by adjusting the pH of chlorous acid water to 6.0 with phosphate buffer (pH5.0). According to the Notification No.15 from the Ministry of Health, Labor, and Welfare Japan [31], chlorous acid water is defined as a solution produced by the following procedure. Saturated NaCl solution was acidified by HCl and electrolyzed in diaphragmless apparatus. The solution is strongly acidified with sulfuric acid to produce chloric acid (HClO_3_). Chlorous acid water (containing 40,000–60,000 ppm HClO_2_) is produced by treating the chloric acid solution with hydrogen peroxide. WACAW and NaClO were diluted with deionized water to adjust to the setting chlorine levels. The pH of each preparation was monitored with pH meter (LAQUAtwin B-72, Horiba Ltd., Japan).

### Spectrophotometric pattern analysis of WACAW and ASC

Chemical composition of WACAW and ASC was compared by spectrophotometric analysis. The FAC level of WACAW and ASC was diluted to 2,400 ppm with deionized water. After the WACAW and ASC were further diluted at 100- and 10-fold with deionized water, these reagents were kept in the dark at 15°C, and the absorbance (200–400 nm) was measured periodically until 7 days after preparation using a spectrophotometer (Hitachi U-5100).

### Plaque assay

The virucidal activity of WACAW and NaClO was evaluated using FCV F9 strain obtained from the ATCC. The FCV was amplified by infecting confluent Crandell Rees feline kidney (CRFK) cell monolayers cultured in a T75 flask in Dulbecco’s minimal essential medium (DMEM) supplemented with 10% FBS. When most of the cells detached from the flask, the supernatant was collected, centrifuged to remove cell debris and divided into aliquots that were stored at -80°C until use as a viral inoculum. The stock FCV viral titer was 2.0 x 10^7^ plaque-forming units (PFU) ml^-1^.

Antiviral activities of the test sanitizers towards FCV were assessed by a standard plaque assay. BSA, polypeptone, meat extract or yeast extract are listed as organic matter for a viral load examination in the guideline for examination of food hygiene in Japan [32]. To evaluate the effect of organic substances on the virucidal efficiency of the test sanitizers, a viral inoculum (FCV titer: 2.0 x 10^6^ PFU ml^-1^) containing BSA (5.0%, w/v), polypeptone (5.0%, w/v) or meat extract (5.0%, w/v) was prepared. The viral inoculum (0.1 ml) was mixed with 0.9 ml saline or test sanitizers. To examine the effect of higher concentration of BSA (5.0% w/v of final concentration), a viral inoculum (FCV titer: 2.0 x 10^6^ PFU ml^-1^) containing 10% BSA (w/v) (0.5 ml) was mixed with an equal amount (0.5 ml) of the test sanitizers, which were adjusted at 2-fold higher FAC to the setting. The mixtures were incubated at 25°C in block incubator (BI-515, ASTEC Co. Ltd., Japan) for 0.5, 1, 3, 5, and 10 min before samples (0.1 ml) were collected and neutralized with 0.9 ml 0.1 M sodium thiosulfate in 0.6 M Tris-HCl (pH 7.0). The neutralized solutions (0.5 ml each) were poured onto a CRFK confluent culture monolayer (grown in 60 mm dishes containing DMEM plus 10% FBS (w/v), 100 U penicillin ml^-1^ and 100 μg ml^-1^ streptomycin, washed with PBS before treatment) and rocked for 1 h in a CO_2_ incubator. After removing the viral solution, the cells were overlaid with 0.5% methylcellulose (w/v) in minimum essential media plus 0.5% FBS (w/v). The treated cells were incubated in a CO_2_ incubator at 37°C with 5% CO_2_ (v/v). After 48 h, the cells were fixed and stained with 0.05% crystal violet (w/v) in a 10% formaldehyde solution (w/v) for 4 h, then washed vigorously with tap water. The clear plaques were enumerated and the PFU/ml of the surviving FCV was calculated.

### Preparation of *C*. *difficile* spores

*C*. *difficile* strain 630 was used in this study. A glycerol stock of the strain was streaked onto a brain-heart infusion with yeast extract and L-cysteine (BHIS) agar plate and anaerobically incubated at 37°C for 72 h in an anaerobic chamber (Forma Scientific) conditioned with mixed gas (N_2_, 80%; H_2_, 10%; CO_2_, 10%, v/v). A single colony was inoculated into 200 ml Gifu anaerobic broth (GAM, Nissui Pharmaceutical Co. Ltd.) and anaerobically incubated at 37°C for 1 week. After spore formation was confirmed by staining with malachite green, the culture was centrifuged at 6,000 rpm for 15 min at 4°C. The pellet was suspended in 5 ml of PBS (pH 7.4), and the suspension was heated at 60°C for 20 min. The spores were collected by centrifugation at 12,000 rpm for 5 min at 4°C and washed three times with 4 ml of sterile distilled water. Spores were finally suspended in 2 ml of saline containing 0.05% Tween 20 and dispersed by brief sonication. Viable spore numbers were determined by plating the dilution of the spore solution with saline containing 0.05% Tween 20 (w/v) on BHIS agar containing the *C*. *difficile* spore germinant taurocholic acid (TCA). The spore suspension was divided into aliquots and stored at -80°C until use. The stock *C*. *difficile* spore titer was 1.2 x 10^6^ colony-forming units (CFU) ml^-1^.

### Sporicidal assay

The sporicidal activities of WACAW and NaClO against *C*. *difficile* 630 spores were compared with or without 0.5% polypeptone (w/v). In assays with polypeptone, polypeptone was added to the spore solution to 5% (w/v) before treatment with the test sanitizers. The spore solution (0.1 ml) was mixed with 0.9 ml of WACAW or NaClO with FAC adjusted to 50, 100 or 200 ppm. The mixture was kept at 25°C in an anaerobic chamber, and 0.1 ml was sampled at 1, 5, and 10 min after incubation. These samples were immediately neutralized with 0.1 M sodium thiosulfate and serially diluted with saline containing 0.05% Tween 20 (w/v). The appropriate dilutions were plated onto BHIS agar containing 0.1% TCA (w/v). After the plates were anaerobically incubated at 37°C for 72 h, the number of colonies was counted.

### Microbicidal assay against FCV and *C*. *difficle* spores in rat feces

The cecal contents of two male germ-free WA/Jic rats (12 weeks old, Clea Japan Inc.) were aseptically collected and stocked at -80°C until use. The cecal contents were weighted and suspended in PBS (pH 7.4) to 10% (w/v). The inoculum was prepared by mixing the FCV or *C*. *difficle* spore stock solution with 10% rat cecal suspension at 1:1. The inoculum (0.1 ml), which included 5.0% (w/v) rat cecal content, was added to the 0.9 ml of test sanitizer (WACAW or NaClO): the final concentration of rat cecal content in the reaction mixture was 0.5% (w/v). The surviving FCV or *C*. *diffcile* spore after 1-min exposure to the test sanitizer was enumerated as described above.

### Monitoring of FAC after partial neutralization with sodium thiosulfate

According to the following chemical equations (upper and lower equation for NaClO and WACAW, respectively), an 8-fold amount of sodium thiosulfate is needed to neutralize WACAW compared with NaClO.

$$4HClO+Na_{2}SO_{3}+H_{2}O\to NaCl+2H_{2}SO_{4}+HCl$$

$$HClO_{2}+2Na_{2}SO_{3}\to 2Na_{2}SO_{4}+HCl$$

Therefore, 1 ml of WACAW (pH6.5) and NaClO (pH10.3) with 1, 200–1,300 ppm FAC was partially neutralized with 336 μl and 42 μl of 0.1 M sodium thiosulfate, respectively. These solutions were kept at 4°C, and their FAC levels were monitored by the DPD method upon periodical sampling.

### Statistical analysis

Data were expressed as mean ± standard deviation. Statistical analysis of the data was performed with StatFlex ver. 6.0 (Artech Co., Ltd., Tokyo) using analysis of variance (ANOVA) followed by Dunnett’s test. Data were considered to be significantly different if the *p* value was less than 0.05.

### Ethics

Animal experiment was carried out in accordance with Japanese legislation and guidelines under the jurisdiction of the Ministry of Education, Culture, Sports, Science and Technology, Japan. The protocols of animal experiments were approved by the committee of animal experiment of the Kagawa University (Approved Number;16666–1). Animal care, housing, feeding, sampling, observation, and environmental enrichment were performed in accordance with the guidelines.

## Results

### Characterization of WACAW and ASC by spectrophotometric analysis

**Fig 1:**
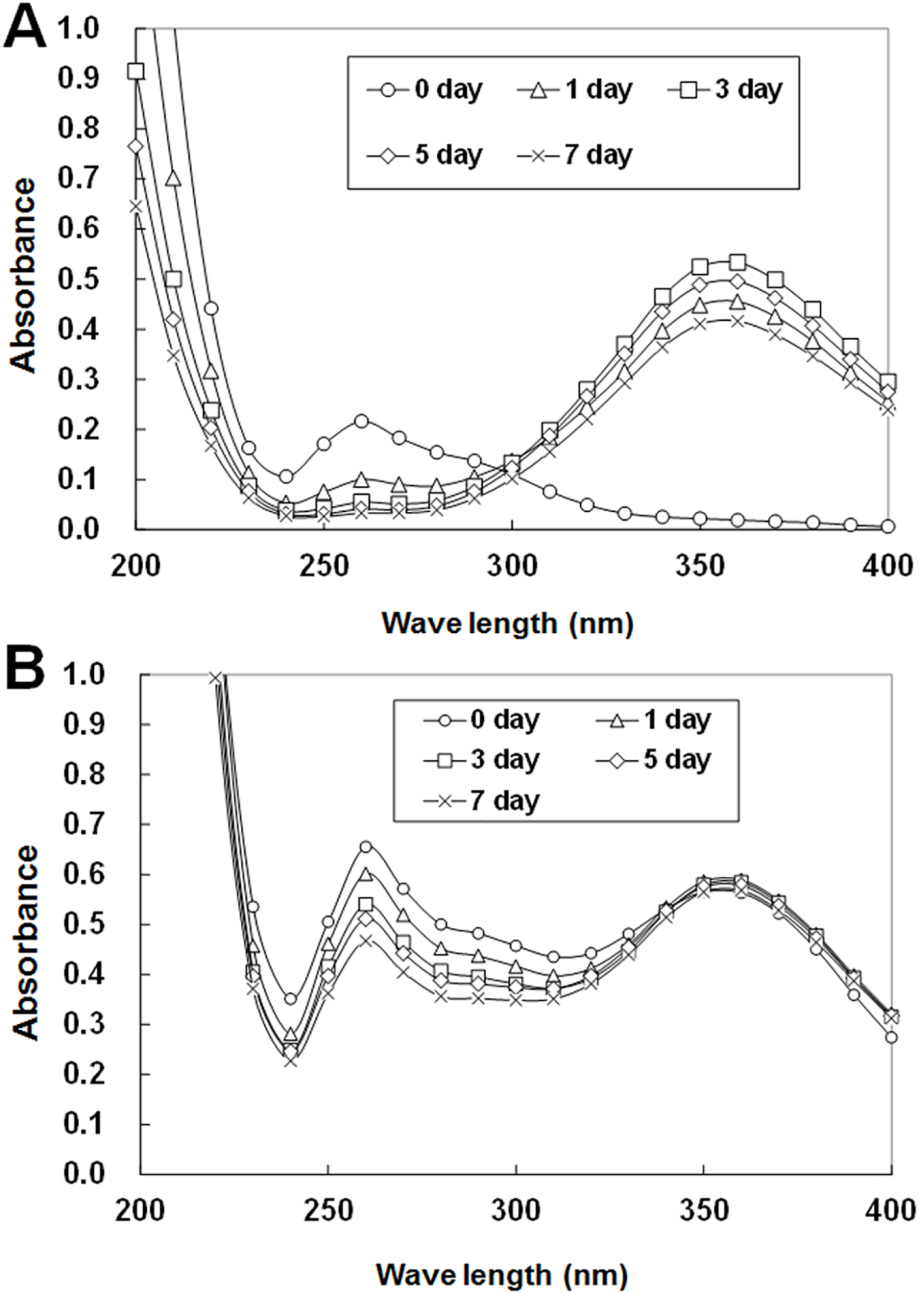
**Spectrophotometric characterizations of ASC (A) and WACAW (B).** Spectra were measured at the indicated day after preparation. The peaks around 260 nm and 360 nm indicate ClO_2_^-^ and ClO_2_, respectively. ASC and WACAW were diluted with distilled water at 10- and 100-fold for the absorbance to be within the measurable range.

Both WACAW and ASC contained various chlorous compounds such as ClO_2_, ClO_2_^-^, and HClO_2_. ClO_2_^-^ and ClO_2_ show maximum absorbance at around 260 nm and 360 nm, respectively. As shown in Fig 1, spectrophotometric patterns were similar between ASC and WACAW immediately after preparation (day 0). However, in the case of ASC, the peak at 260 nm decreased and the 360 nm peak showing ClO_2_ increased periodically (Fig 1A). In contrast, the spectrophotometric pattern of WACAW was similar to that on day 0 even 7 days after preparation, indicating that the chemical composition of WACAW is more stable (Fig 1B).

### FCV inactivation by WACAW and NaClO in the presence and absence of BSA

**Fig 2:**
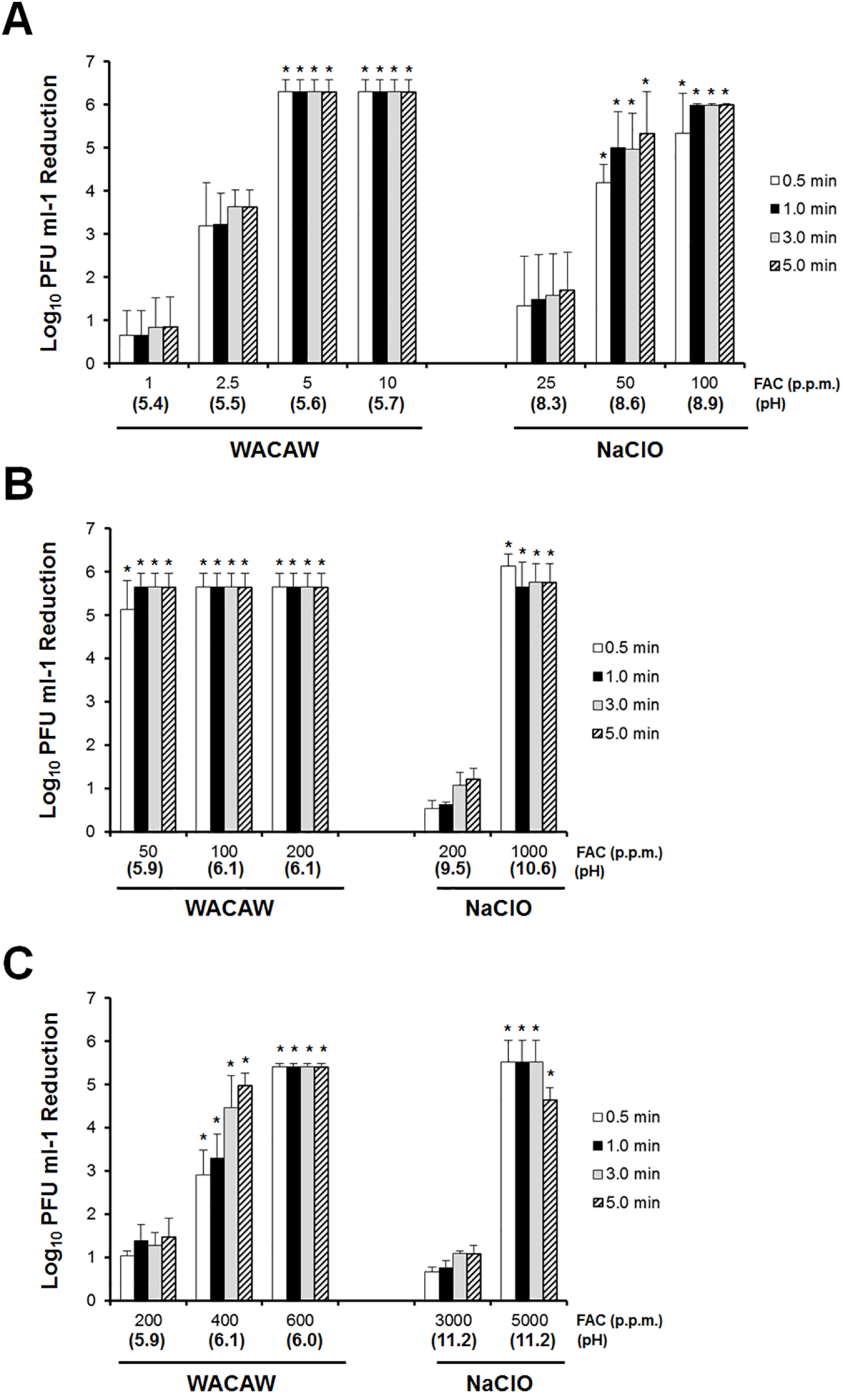
Effect of BSA on FCV inactivation by WACAW and NaClO. The virucidal activity against FCV F9 was examined without BSA (A) or with 0.5% (w/v) BSA (B) or 5.0% (w/v) BSA (C). The remaining FCV infectivity after treatment with the test sanitizers for the indicated times was quantified by plaque assay. Data are expressed as means ± standard deviations obtained from three independent experiments. * Significantly different from the data in NaClO with 25 ppm (A), 200 ppm (B) and 3,000 ppm (C) at each time point (*p* <0.01).

The inactivation of FCV by WACAW and NaClO was tested via plaque assay. In the absence of BSA, WACAW at 5 ppm FAC and NaClO at 50 ppm FAC decreased FCV over 4 log_10_ (Fig 2A). In the presence of 0.5% BSA (w/v), NaClO at 200 ppm FAC showed less virucidal activity and achieved only 1 log_10_ decrease even after a 5 min treatment (Fig 2B). NaClO at 1,000 ppm FAC was needed to achieve a >5 log_10_ FCV decrease within 30 sec. In contrast, WACAW achieved a >5 log_10_ decrease in FCV at 50 ppm FAC within 30 sec even in the presence of 0.5% BSA (w/v). In the presence of 5.0% BSA (w/v), NaClO at 3,000 ppm FAC only decreased 1 log_10_ FCV even after 5 minutes of treatment, and NaClO at 5,000 ppm FAC was needed to decrease >5 log_10_ FCV within 30 sec (Fig 2C). In the case of WACAW, 200 ppm FAC was not sufficient for FCV inactivation, but WACAW at 400 ppm FAC reduced FCV more than 4 log_10_ within 3 min. In addition, WACAW at 600 ppm FAC reduced >5 log_10_ FCV within 30 sec. These data indicate that WACAW is more effective for FCV inactivation than NaClO in the presence of BSA.

### FCV inactivation by WACAW and NaClO in the presence of polypeptone

**Fig 3:**
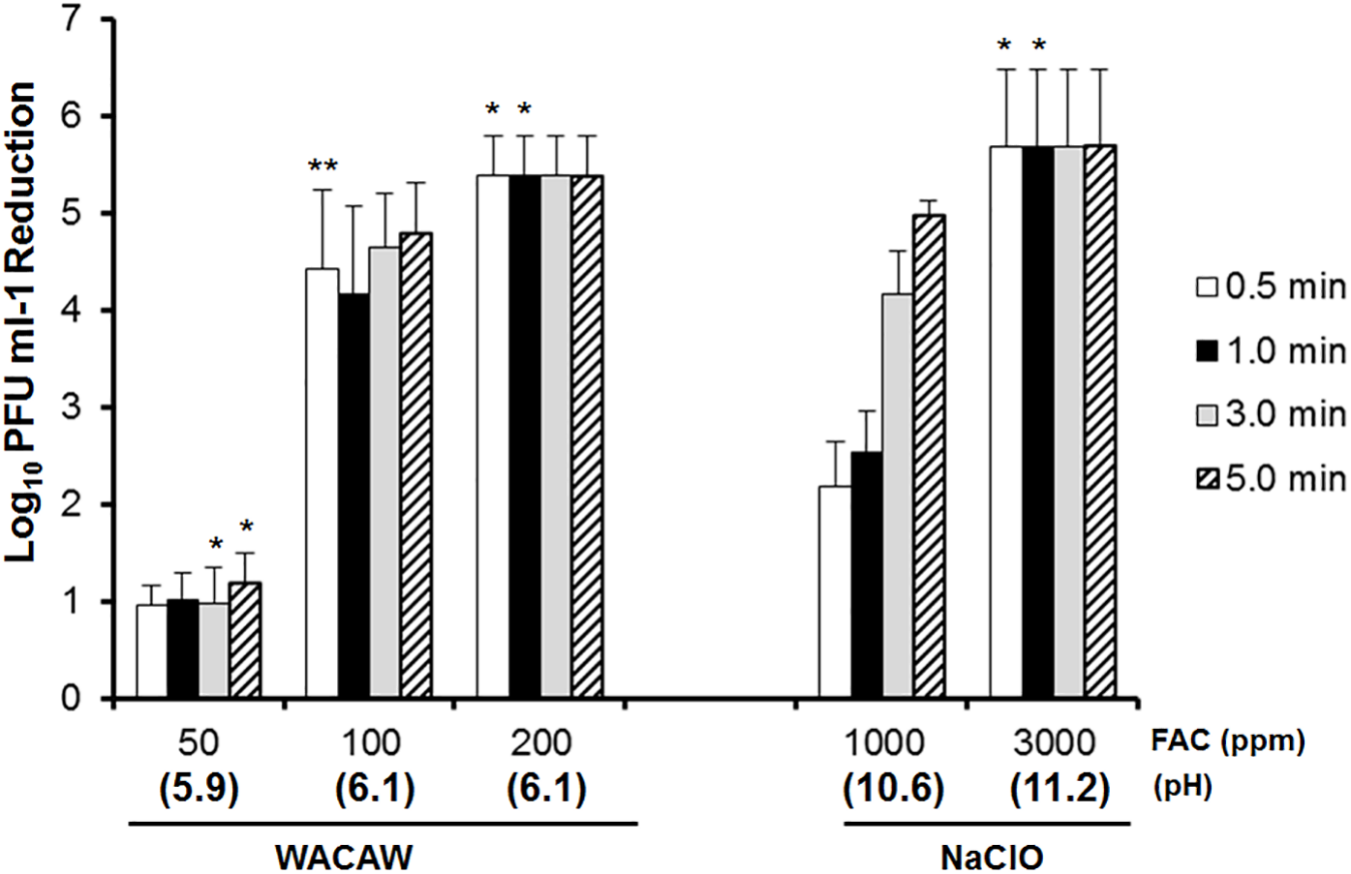
Effect of polypeptone on FCV inactivation by and WACAW and NaClO. The virucidal activity against FCV F9 was examined in reaction mixtures containing 0.5% polypeptone (w/v). The remaining FCV infectivity after treatment with the test sanitizers for the indicated times was quantified by plaque assay. Data are expressed as means ± standard deviations obtained from three independent experiments. Significant difference from the 1,000 ppm NaClO at each time point was expressed by * (*p* <0.01) or ** (*p* <0.05).

Polypeptone (0.5%, w/v) was used to increase the protein load in the FCV inactivation assay. As shown in Fig 3, polypeptone interfered with the virucidal activity of WACAW and NaClO against FCV to a greater extent than BSA. NaClO at 1,000 ppm FAC resulted in a 4 log_10_ FCV decrease with the 3-min treatment, and 3,000 ppm FAC in NaClO inactivated >5 log_10_ FCV within 30 sec. In the case of WACAW, the FAC level required to decrease >5 log_10_ FCV within 30 sec was 200 ppm.

### FCV inactivation by WACAW and NaClO in the presence of meat extract

**Fig 4:**
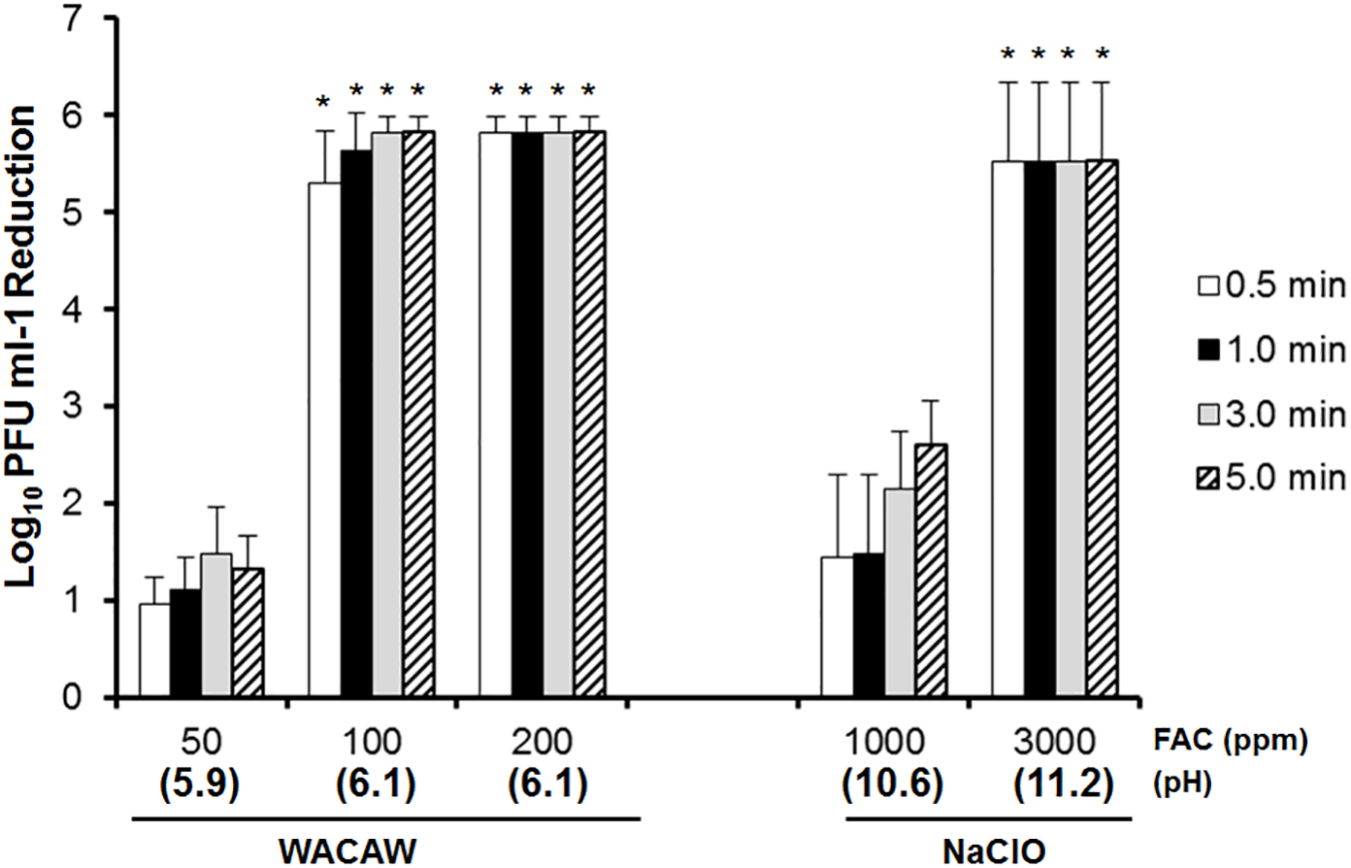
Effect of meat extract on FCV inactivation by WACAW and NaClO. The virucidal activity against FCV F9 was examined in reaction mixtures containing 0.5% meat extract (w/v). The remaining FCV infectivity after treatment with the test sanitizers for the indicated times was quantified by plaque assay. Data are expressed as means ± standard deviations obtained from three independent experiments. Significant differences from the 1,000 ppm NaClO at each time point were expressed by * (*p* <0.01).

Meat extract (0.5%, w/v) was also tested to increase the protein load in the FCV inactivation assay. Interestingly, meat extract affected the virucidal activity of NaClO to a greater extent than polypeptone, while the effect of the meat extract on the virucidal activity of WACAW was weaker than polypeptone. As shown in Fig 4, NaClO at 1,000 ppm FAC achieved less than a 3 log_10_ FCV decrease even after 5-min treatment. As with 0.5% polypeptone (w/v), NaClO at 3,000 ppm FAC was needed to reduce >5 log_10_ FCV within 30 sec. Although WACAW at 50 ppm FAC did not show virucidal activity (only 1 log_10_ FCV decrease), WACAW at 100 ppm and 200 ppm FAC achieved >5 log_10_ FCV decrease within the 30-sec treatment.

### *C*. *difficile* spore inactivation by WACAW and NaClO

**Fig 5:**
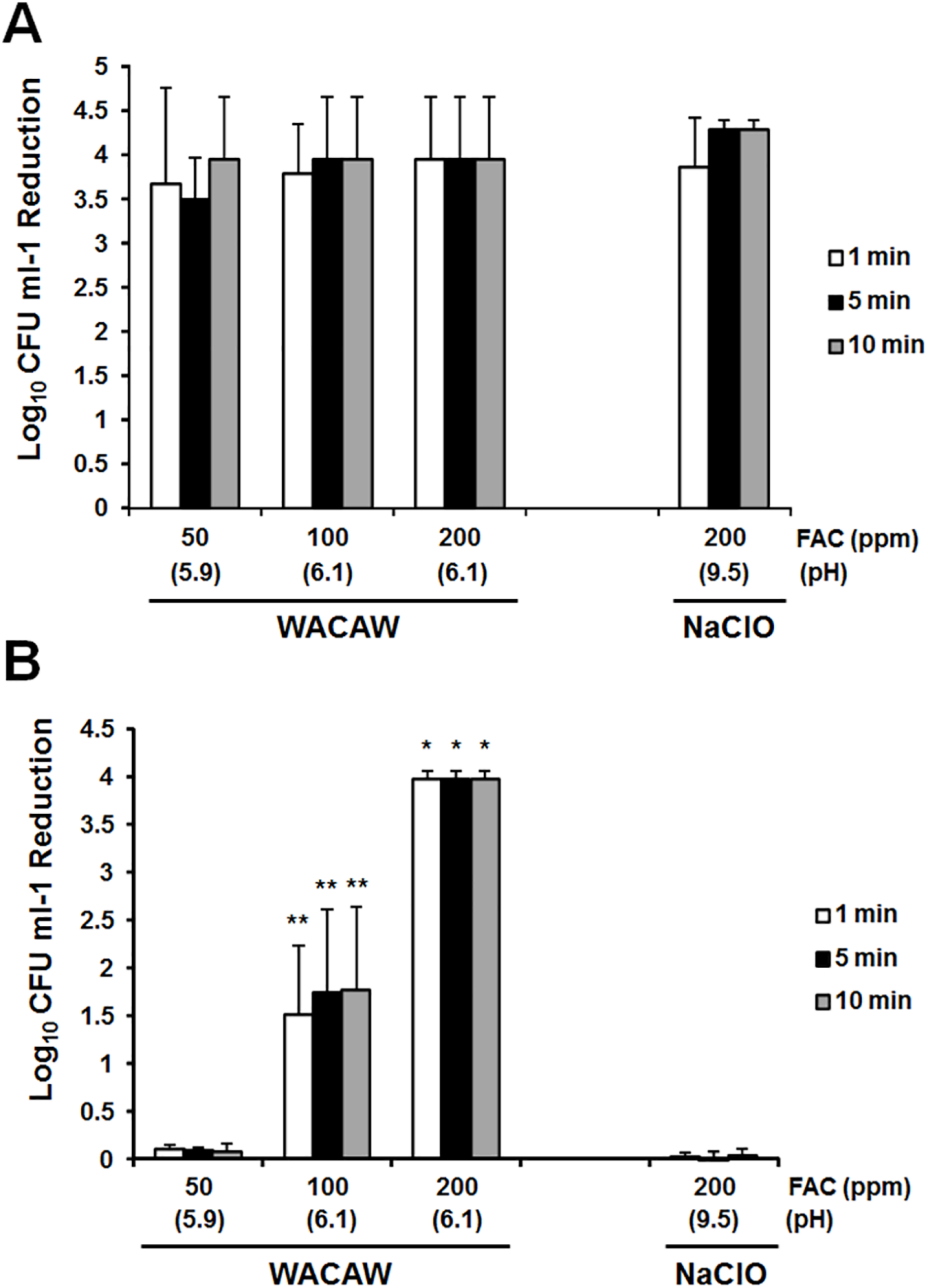
*C*. *difficile s*pore inactivation by WACAW and NaClO. The sporicidal activity against *C*. *diffcile* 630 spores was examined in reaction mixtures without (A) or with 0.5% (w/v) polypeptone (B). The remaining viable *C*. *difficile* spores after treatment with the test sanitizers for the indicated times were quantified using a standard plate assay with BHIS supplemented with taurocholic acid. Data are expressed as means ± standard deviations obtained from three independent experiments. Significant differences from the 200 ppm NaClO at each time point were expressed by * (*p* <0.01) or ** (*p* <0.05).

The sporicidal activity of WACAW against *C*. *difficile* 630 spores was examined. In the absence of additional protein, WACAW at 50, 100, and 200 ppm FAC inactivated >3.5 log_10_ *C*. *difficile* spores within 1 min, and NaClO at 200 ppm FAC showed equivalent sporicidal activity (Fig 5A). However, in the presence of 0.5% polypeptone (w/v), no sporicidal activity on *C*. *difficile* spores was observed with NaClO at 200 ppm FAC whereas WACAW at 200 ppm FAC decreased >3.5 log_10_ *C*. *difficile* spores within 1 min under the same conditions (Fig 5B). WACAW at 100 ppm FAC also showed weak sporicidal activity (1.51–1.77 log_10_ CFU decrease) on *C*. *difficile* spores in the presence of 0.5% polypeptone (w/v).

### Inactivation of FCV and *C*. *difficile* spore in rat cecal content

**Fig 6:**
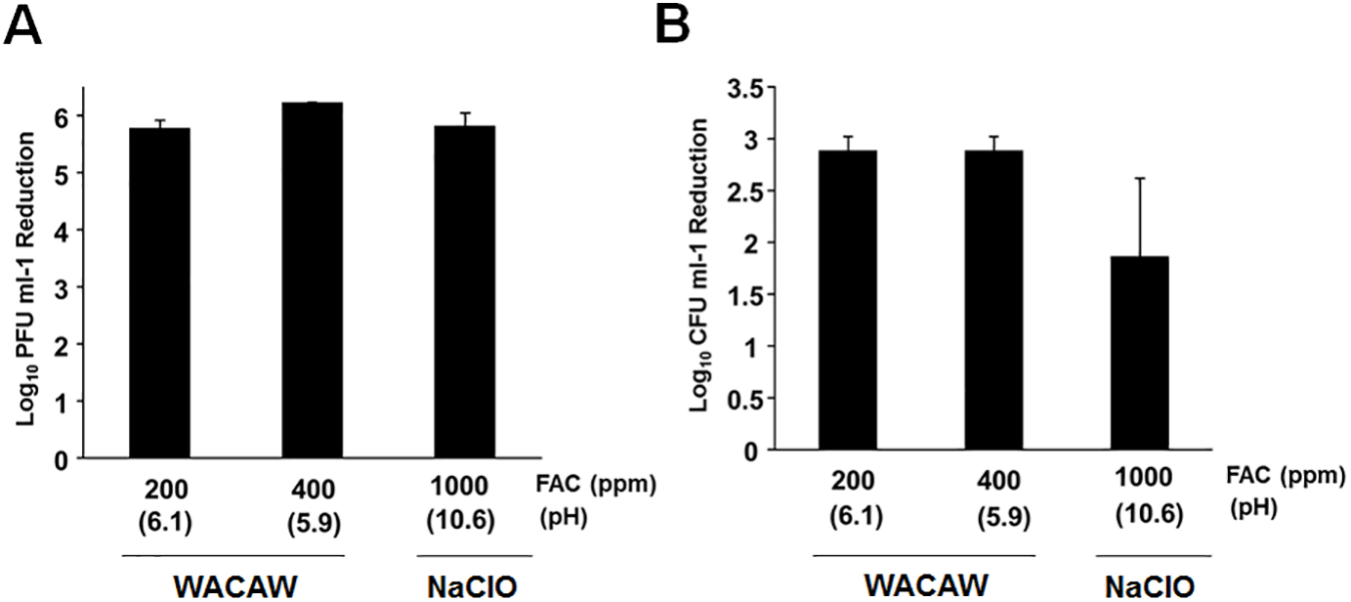
**Inactivation of FCV (A) and *C*. *difficile s*pore (B) in the presence of rat cecal content.** The sporicidal activity against *C*. *diffcile* 630 spores was examined in reaction mixtures containing 0.5% (w/v) rat cecal content. The remaining viable *C*. *difficile* spores after treatment with the test sanitizers for the indicated times were quantified using a standard plate assay with BHIS supplemented with taurocholic acid. Data are expressed as means ± standard deviations obtained from three independent experiments. No significant difference was found.

Fecal-oral route plays an important role in the human to human transmission of norovirus and *C*. *difficile* spores. To simulate the disinfection in practical condition, rat cecal content was used as an organic matter load. As shown in Fig 6, 200 ppm and 400 ppm WACAW inactivated FCV and *C*. *difficile* spore at equivalent or superior levels to those by 1,000 ppm NaClO.

### Monitoring of FAC after neutralization with sodium thiosulfate

**Fig 7:**
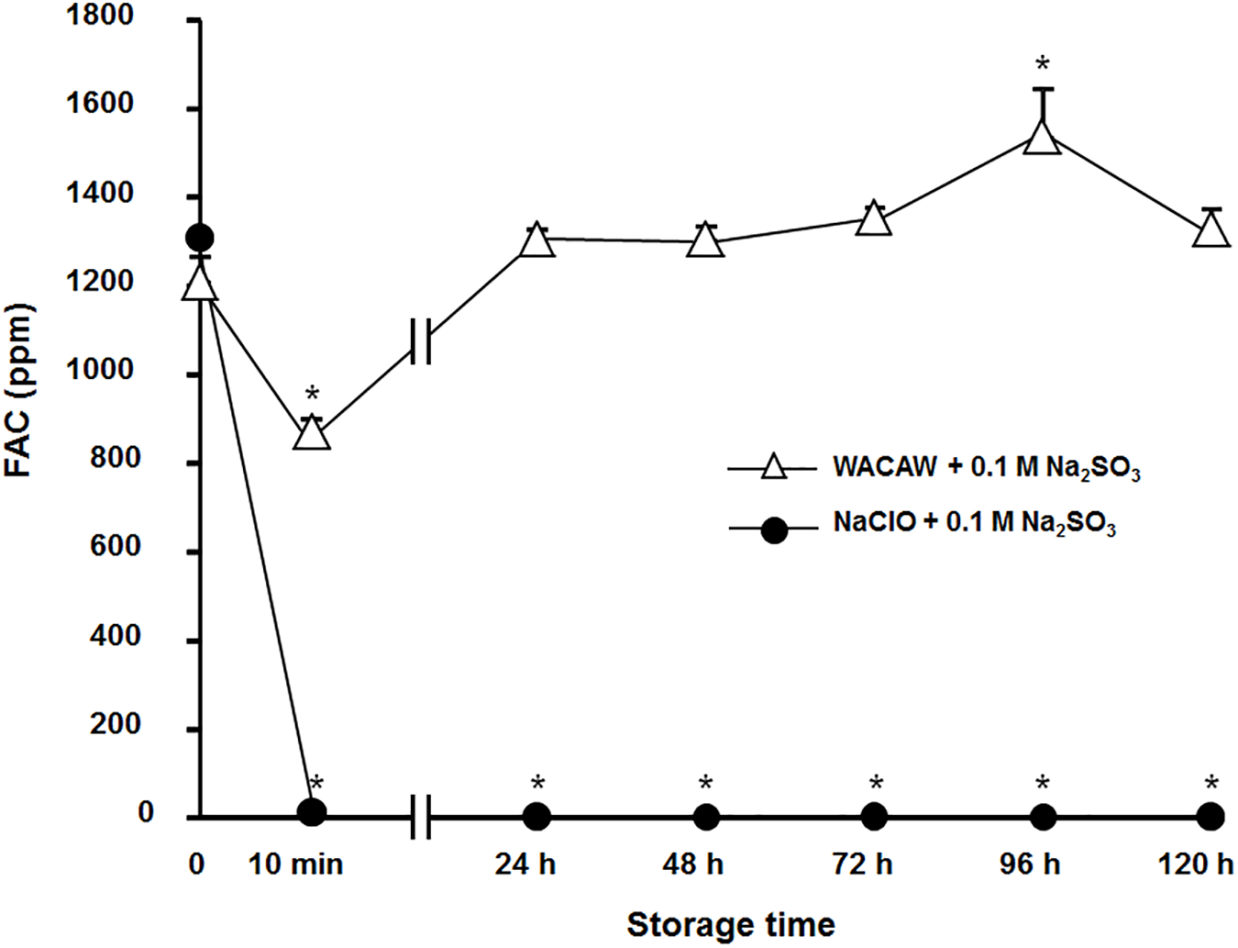
Monitoring of FAC after neutralization with sodium thiosulfate. After WACAW and NaClO, adjusted to FAC levels of 1,300 ppm, were partially neutralized with 0.1 mM sodium thiosulfate (Na_2_SO_3_), the change of the FAC levels were monitored at the indicated time points by the *N*,*N*-diethyl-*p*-phenylenediamine sulfate method. The means and standard deviations of three experiments are shown. Significant differences from the initial FAC level were expressed by * (*p* <0.01).

After 1,300 ppm FAC each of NaClO and WACAW was partial neutralized with 0.1 M sodium thiosulfate, their FAC levels were monitored periodically over 5 days. The 336 μl and 42 μl of 0.1 M sodium thiosulfate were calculated to neutralize a half of FAC of 1,300 ppm WACAW and NaClO, respectively. As shown in Fig 7, the FAC level of WACAW (1 ml) immediately decreased to 820 ppm when 336 μl of 0.1 M sodium thiosulfate was added. Interestingly, the FAC level of partially neutralized WACAW was restored to its initial level within 24 h after neutralization. In contrast, the FAC level of the NaClO (1 ml) was rapidly decreased to around 10 ppm when 42 μl of 0.1 M sodium thiosulfate was added, and its FAC level did not recover during the observation period.

### Monitoring of combined chlorine (CMC) level after contact with BSA

Chlorine-based sanitizers often generate chlorinated substances such as chloramines (e.g. NH_2_Cl). These combined form of chlorine are less reactive and quantified as combined chlorines (CMC). As shown in Table 1, a large amount of chlorine atom in WACAW was detected as CMC due to the high content of ClO_2_^-^. On the other hand, the chlorine atoms in NaClO were all reactive FAC. After 2,000 or 4,000 ppm TC of the test sanitizer (WACAW or NaClO) was exposed to 0.5% BSA for 10 min, the changes in TC, FAC and CMC levels were monitored. The TC levels of NaClO was decreased by 46–61% while those of WACAW remained 87–88%. The FAC of WACAW (4,000 ppm TC) or NaClO (4,000 ppm TC) decreased to 9.0% and 0.05%, respectively. Interestingly, the CMC levels were markedly increased in NaClO after contact with BSA while no increase in CMC was observed in WACAW. These results indicate that WACAW does not chlorinate the target substances while NaClO produces a large amount of chlorinated substances after contact with organic matters.

**Table 1:** Changes of TC, FAC and combined chlorine levels in the test sanitizers after contact with BSA.

Sanitizers (setting TC)	TC		FAC		CMC	
	Before	After	Before	After	Before	After
WACAW (2,000 ppm TC)	1921.2±48.4	1677.9±80.8	108.11±1.8	0.7±0.2	1813.1±47.0	1677.2±80.6
WACAW (4,000 ppm TC)	3671.1±12.1	3215.5±93.7	228.68±12.1	20.6±2.6	3442.5±12.1	3194.9±93.7
NaClO (2,000 ppm TC)	2002.0±20.7	1088.7±88.2	2091.67±108.2	13.6±0.8	<0.00	1075.0±88.3
NaClO (4,000 ppm TC)	3913.9±50.5	1507.1±126.2	4391.08±99.5	24.3±0.7	<0.00	1482.8±126.2

Abbreviation: TC, total chlorine; FAC, free available chlorine, CMC, combined chlorine.

## Discussion

NaClO is a highly reactive, chlorine-based disinfectant that shows a wide antimicrobial spectrum that includes bacterial spores and non-enveloped viruses. Since *C*. *difficle* spore and FCV, a human norovirus surrogate, are resistant to the commonly used disinfectants, environmental sanitation with NaClO is applied to block the transmission of these pathogens [33, 34]. Park et al. reported that alcohol (67–79%, pH4.1–7.4) achieved less than 1.0 log_10_ reduction in FCV F9 strain [35]. In addition, Lawrey et al. demonstrated that 70% ethanol has no sporicidal activity against *C*. *difficile* 630 spore [36]. In this study, WACAW with >5 and >50 ppm FAC showed >5.0 log_10_ and >3.0 log_10_ reduction in FCV F9 and *C*. *difficile* 630 spore, respectively, in the absence of organic substances. These results indicate that WACAW is an effective sanitizer to control the outbreak of norovirus and *C*. *difficle* infections.

In the practice of disinfection of environmental surfaces contaminated with human vomit or stool, one must consider that disinfectants rapidly lose its antimicrobial activity in contact with organic matter. Indeed, it has been reported that 10,000 ppm NaClO failed to inactivate *Staphylococus aureus* in human blood [37]. WACAW was also required higher FAC or longer treatment to inactivate the FCV and *C*. *difficile* spore in the presence of BSA, polypeptone or meat extract (Figs 2–5). However, WACAW inactivated FCV and *C*. *difficile* spore at much lower FAC level than NaClO in the presence of organic materials. In addition, WACAW (4,000 ppm TC) retained 9.0% of the initial FAC after 10-min contact with 0.5% BSA while the FAC of NaClO (4,000 ppm TC) decreased to 0.05% (Table 1). These results indicate that WACAW is more stable than NaClO under organic matter-rich condition.

ASC has been reported to be relatively stable and have a bactericidal effect on foods superior to NaClO under organic matter-rich conditions, such as elimination of *Salmonella* or pathogenic *Escherichia coli* from vegetables or meat [20, 25]. Therefore, ASC is now widely used for sanitation of foods and environmental surfaces. ASC (pH 2.3–2.9) is prepared with sodium chlorite (NaClO_2_) and strongly acidified with acids generally recognized as safe, such as citrate or lactate. ASC rapidly produces a large amount of chlorous acid (HClO_2_) that converts to ClO_2_ gas (released into the air) under strongly acidic conditions [28]. Therefore, its bactericidal activity is short-lived, and ASC must be prepared at the time of use. We have recently reported that WACAW not only retains its microbicidal activity but is also stable under organic matter-rich conditions [26]. Since the microbicidal effect of WACAW is dependent on the oxidative potential of HClO_2_ and ClO_2_ [28], the killing mechanisms of ASC and WACAW are thought to be similar. However, the spectrophotometric pattern of WACAW seemed to be stable while ClO_2_ became dominant in ASC time-dependently. The relative tolerance of WACAW to organic matter might be derived from the stability of its chemical composition.

In this study, we tested three kinds of proteinaceous compounds including BSA, polypeptone and meat extract, which simulate blood, vomit and feces, to evaluate the stability of test sanitizers. The results showed that the effect on microbicidal activity of WACAW and NaClO was markedly different among the compounds. Polypeptone and meat extract inhibited the microbicidal activity of WACAW and NaClO to FCV and *C*. *difficile* spores to a greater extent than BSA. Although BSA is widely used to evaluate the stability under organic matter-rich conditions, our results indicate that the disinfection efficacy of sanitizers must be examined under various organic matter loads.

Interestingly, different modes of interference on the microbicidal activities of WACAW and NaClO were observed among BSA, polypeptone and meat extract. Measurement of the protein concentrations of 0.5% polypeptone (w/v) (1.12 mg ml-1) and 0.5% meat extract (w/v) (1.03 mg ml^-1^) indicates that they contain many organic substances other than proteins. Interestingly, WACAW and NaClO showed different responses to these organic substances. The virucidal activity of NaClO was more affected by meat extract than polypeptone, while the activity of WACAW was more strongly decreased by polypeptone than BSA and meat extract, indicating that the reaction selectivity of WACAW and NaClO is different. In fact, Ingram *et al*. reported that HClO_2_-based sanitizers preferentially react with sulfhydryl groups [22, 38]. However, at present, it is unknown which chemical composition in polypeptone is involved in the inhibition of the virucidal activity of WACAW. Identification of preferential active groups for WACAW might lead to the discovery of antimicrobial agents that selectively react with FCV capsid proteins and *C*. *difficile* spore coat proteins but do not react with organic matter in human excretions.

Although how WACAW could retain its microbicidal activity was unclear, we hypothesize that this stability came from the large dissociation of total chlorine level and FAC level in WACAW (the total chlorine level is nearly 10-fold higher than FAC). WACAW mainly contains three kinds of oxygenated chlorines: HClO_2_, ClO_2_^-^ and ClO_2_. Of these, ClO_2_^-^ shows weak oxidative potential, making only a minor contribution to FAC level or antimicrobial killing. Therefore, ClO_2_^-^ content is considered to be much higher than HClO_2_ and ClO_2_ in WACAW. This ClO_2_^-^ might continuously supply HClO_2_ under acidic conditions even after FAC is consumed from contact with microorganisms or organic matter. As shown in Fig 7, FAC decreased immediately after neutralization of NaClO and WACAW with sodium thiosulfate. However, in the case of WACAW, FAC was restored to the original level until 24 h after neutralization; such recovery was not observed for NaClO.

The data shown in Table 1 clearly demonstrated the difference in oxidation mechanism of WACAW and NaClO, where the CMC increased after NaClO being exposed to BSA while the CMC level did not increase in WACAW. NaClO chlorinated organic substances but WACAW probably does not. NaClO is known to produce mutagenic compounds such as trihalomethane during oxidation of organic substances [39, 40]. From the viewpoint of food safety, the finding indicates that WACAW might be a suitable sanitizer in food industries.

## Conclusions

WACAW exhibited microbicidal activity superior to NaClO against FCV and *C*. *difficile* spores even in the presence of BSA (0.5% or 5.0%, w/v) or 0.5% (w/v) each of polypeptone or meat extract. WACAW may be a useful sanitizer to inactivate the non-enveloped viruses or bacterial spores present in foods and on environmental surfaces contaminated with human excretions.
